# Quality of life in keratoconus: evaluation with Keratoconus Outcomes Research Questionnaire (KORQ)

**DOI:** 10.1038/s41598-021-92346-1

**Published:** 2021-06-21

**Authors:** Roberto Damian Pacheco Pinto, Ricardo Yuji Abe, Flávia Cid Gomes, Paulo Rodolfo Tagliari Barbisan, Alexandre Fattah Martini, Daniel de Almeida Borges, Arthur Gustavo Fernandes, Carlos Eduardo Leite Arieta, Monica Alves

**Affiliations:** 1 Department of Ophthalmology and Otorhinolaryngology, University of Campinas, Campinas, São Paulo Brazil; 2 Departamento de Oftalmologia e Ciências Visuais, Escola Paulista de Medicina, Universidade Federal de São Paulo - UNIFESP, São Paulo, SP Brazil

**Keywords:** Corneal diseases, Eye manifestations, Quality of life

## Abstract

To assess the quality of life of keratoconus patients using the Keratoconus Outcomes Research Questionnaire (KORQ), translated and validated in Portuguese language. The KORQ is the only validated keratoconus specific questionnaire and has a high rating for its psychometric properties. This cross-sectional study enrolled 100 keratoconus patients from a tertiary referral eye hospital between April 2018 and June 2019. Associations between age, sex, allergic conjunctivitis, keratoconus stage, best-corrected visual acuity (BCVA), maximum simulated keratometry (Kmax), steep keratometry (K2), pachymetry, treatments performed, hydrops, and KORQ scores were evaluated using univariate (Wilcoxon test and the Kruskal Wallis test) and multivariate linear regression with stepwise backward modeling. Lower KORQ scores are associated with better quality of life, whereas, higher scores are associated with greater impairment of functional activities and symptoms. Among the 100 patients, mild, moderate, and severe keratoconus were observed in 15%, 46% and 39% of participants, respectively. Univariate analysis showed lower function scores values, with male sex (*p* < 0.05) and both functional and symptom scores were significantly associated with BCVA < 0.3 (LogMAR) (*p* < 0.05). Multivariate analysis indicated significantly lower functional scores in individuals with BCVA < 0.3 (LogMAR) (*p* < 0.001) and those with a history of crosslinking treatment (*p* = 0.022), while symptom scores were only significantly associated with only BCVA < 0.3 (LogMAR) (*p* < 0.001). In patients with keratoconus, BCVA in the better eye and history of crosslinkig were factors associated with better quality of life scores using the KORQ.

## Introduction

Keratoconus it is a progressive corneal disease with a typical onset in adolescence or early adulthood, although some cases of severe keratoconus have been reported in children as young as 4 years of age^1^. It is a progressive asymmetric corneal disease characterized by steepening and distortion, apical thinning, and central corneal scarring^2^. After onset, keratoconus can progress its steepening and increase corneal parameter irregularities, consequently worsening vision^3^. Treatment of keratoconus consists of spectacles, contact lenses, intrastromal corneal ring implants and corneal collagen crosslinking^4^, and when these treatment options are no longer effective for vision rehabilitation, lamellar or penetrating keratoplasty can be performed^5^.

Increasing attention has been given to the assessment of health-related quality-of-life (QoL) outcome measures in clinical trials during the past three decades. Among these measures, vision-related quality-of-life (VQRL) is a person**’**s satisfaction with visual function and how visual ability affects life^6^. Keratoconus significantly impacts on VRQL with a substantial number of patients experiencing a decline in their VRQL over time^6,7^. Even the simplest rehabilitation methods such as spectacles or contact lenses fit can affect QoL due to their negative effects on cosmesis and/or handling inconvenience^8–10^.

Questionnaires have been increasingly implemented as a tool to assess the QoL related to a specific disease, quantify symptoms, evaluate the disease natural course and determine the impact of treatment strategies. However, generic or ophthalmic patient-reported outcome (PRO) measures that are not specific to keratoconus may not include essential items that capture unique, keratoconus-specific QoL issues^3,11^. In fact, studies regarding keratoconus QoL impact have used PRO instruments developed for other conditions, such as for cataract or refractive errors^12^, once there was no keratoconus-specific PRO instrument. An accurate measurement of QoL requires high-quality PRO measures. A recent study evaluating keratoconus QoL using existing questionnaires concluded that this aspect of the disease would be better evaluated by using a QoL questionnaire specifically designed for keratoconus^13^. Fortunaly, a recent psychometrically robust and valid instrument to assess the impact of keratoconus on activity limitation and symptoms was created, called Keratoconus Outcomes Research Questionnaire (KORQ)^14^.

A recent study evaluated psychometric properties of the KORQ through the classical test theory and Rasch analysis and concluded that KORQ is a psychometrically robust PRO measure for QoL parameters evaluation in keratoconus individuals and is appropriate for clinical use and in research^15^ Additionally, another study comparing QoL questionnaires already used for keratoconus concluded that KORQ was the only validated keratoconus specific questionnaire, garnering the highest rating for psychometric properties among all the questionnaires^16^. Thus, KORQ is the recommended **“**gold standard**”** method for development and validation of a patient-reported outcome measures^17^.

Despite these developments, the following important issues regarding to QoL and disease impact remain to be adderssed: (1) there is no previous study evaluating keratoconus QoL keratoconus with the application of KORQ in a group of patients and (2) QoL questionnaire misuse can lead to misleading results, adding confusion and causing a negative contribution to the understanding of the disease impact. Thus, the aim of the present study was to evaluate the QoL in keratoconus patients, using KORQ.

## Methods

The KORQ comprises 2 scales tool, 18 items in the **“**Activity Limitation**”** scale (Table 1) and 11 items in the **“**Symptoms**”** scale (Table 2). Each item has a 4-point rating scale with an additional **“**not applicable**”** option. The patient's score was obtained using a ready-to-use Microsoft Excel scoring (available at http://links.lww.com/OPX/A287 and http://links.lww.com/OPX/A288) spreadsheets for the two KORQ scales. These spreadsheets can be used to convert respondents’ raw scores into person measures in logits without having to run Rasch analysis when the study sample is similar to the original study. Each spreadsheet consists of three sheets labeled as ‘‘rawdata,’’ ‘‘raschscore,’’ and ‘‘raw to Rasch conversion.’’ In these sheets, users are required to register the respondents’ responses to the items with numerical labels (i.e. 1–4) in the ‘‘rawdata’’ sheet, wherein the corresponding Rasch scores automatically appears in the ‘‘raw to rasch conversion’’^14^. Person measure data were rescaled from the original logit scale, using values ranging from 0 to 100, to better understand its meaning. Notably, lower scores are associated with better QoL, whereas higher scores are associated with greater impairment of functional activities and symptoms. In this study, the inclusion criteria was participants aged 18 years and older with a previous keratoconus diagnosis or those who underwent penetrating keratoplasty for keratoconus. Participants with other ocular comorbidities, significant systemic disease, or inability to read Portuguese and understand the questionnaire were excluded. Regarding the Portuguese translation, the process of cross‑cultural adaptation and validation was carried out following the method proposed by Beaton and Gjersing^18,19^. The initial translation of the English version to the Portuguese language was performed by two independent native speaker translators, followed by an interdisciplinary panel evaluation of the translated version. Afterwards, the Portuguese version underwent back translation into English by two independent native speakers, followed by evaluation and comparison with the original English version by the same interdisciplinary panel. For subsequent validation, the translated questionnaire was applied at two different times to a population of 30 subjects, and the results were compared using a concordance analysis. There was a high-correlation coefficients obtained upon comparing the initial application with the results of the questionnaire re-administratered to a sample of 30 individuals indicating excellent concordance in terms of results, repeatability, and reliability of the KORQ Portuguese version^20^. The KORQ was then applied in this study using its final Portuguese version.

**Table 1:** KORQ questionnaire Part I—Activity limitation.

1. How much does your vision interfere with using a computer screen?	10. How much do on coming lights interfere with your ability to see, to do your tasks?
2. How much does your vision interfere with driving during the day?	11. How much does your vision interfere with doing fine tasks at near?
3. How much does your vision interfere with driving during the night?	12. How much does your vision interfere with doing your hobby?
4. How much does your vision interfere with reading street signs?	13. How much does your vision interfere with recognizing faces?
5. How much does your vision interfere with watching TV?	14. How much does your vision interfere with seeing in poor light?
6. How much does your vision interfere with walking up/down steps?	15. How much does your vision interfere with doing household tasks? (e.g. cleaning, ironing, washing, washing up)
7. How much does your vision interfere with avoiding objects in your path?	16. How much does your vision interfere with judging depth?
8. How much does your vision interfere with your ability to do your job?	17. How much does your vision interfere with seeing small objects in the distance? (e.g. golf ball, darts)
9. How much does your vision interfere with seeing in the distance?	18. How much does your vision interfere with sighting tasks? (e.g. camera, microscope, binoculars etc.)

*KORQ* Keratoconus Oucomes Research Questionnaire.

**Table 2:** KORQ questionnaire Part II—Symptoms.

1. How much are you troubled by distorted vision?	7. How much are you troubled by windy days?
2. How much are you troubled by glare and wearing sunglasses all the time?	8. How much are you troubled when you are tired?
3. How much does a bright sunny day interfere with your ability to see, to do your tasks?	9. How much are you troubled by dry days?
4. How much are you troubled by wearing rigid gas permeable contact lenses?	10. How much are you troubled by dusty days?
5. How much are you troubled by headaches when wearing your glasses/contact lenses?	11. How much are you troubled by smoky environments?
6. How much are you troubled by dry eyes?	

*KORQ* Keratoconus Oucomes Research Questionnaire.

This study was conducted in the Department of Ophthalmology at the University of Campinas and was performed in accordance with the tenets of the Declaration of Helsinki, after receiving approval from the Faculty of Health Sciences Ethics Committee. Also, an informed consent form was obtained from all participants. Consecutive keratoconus patients were enrolled from April 2018 to June 2019 from the primary and secondary services. Upon enrollment in the registry, baseline parameters, such as demographic data, ocular history, and prior intervention(s), including previous cross-linking, best-corrected visual acuity (BCVA), steepest keratometry of the central 3 mm of the anterior corneal surface (K2), maximum simulated keratometry (Kmax), and pachymetry, were recorded for each eye. Keratometry and pachymetry were performed using Oculus Pentacam ® (Oculus GmbH, Wetzlar, Germany).

Data analyzed in this study included age, sex, history of allergy and use of eye drops, Best Corrected Visual Acuity (BCVA), keratometry, and pachymetry in the better and worse eye. BCVA was measured with a standardized protocol using Snellen acuity charts, and the results were converted to a LogMAR score. Keratoconus was classified by severity based on the Kmax in the better eye, as follows: mild (Kmax < 48 D), moderate (Kmax 48**–**55 D), and severe (Kmax > 55 D).

Data were analyzed using Stata/SE Statistical Software, Release 14.0, 2015 (Stata Corp, College Station, Texas, USA; www.stata.com). Frequency tables were used for descriptive analysis, and demographic and clinical variables were categorized based on a previous study, which also investigated QoL in keratoconus patients^13^ (male or female, ≤ 27 or > 27 years old, BCVA ≤ 0.3 or > 0.3 (LogMAR), steep K ≤ 52.0 diopters [D] or** > **52.0 [D], central corneal thickness [CCT] ≤ 450 or > 450 μm, hydrops (at least one eye) or not, contact lens wear or not, history of corneal transplant or not, history of Intra corneal ring segment (ICRS) or not, history of corneal crosslinking (CXL) or not, surgical treatments or not). Univariate and multivariate analysis were also performed to investigate factors associated with functional and symptom scores. The multiple linear regression model was determined according to the stepwise backwards criteria and correlations between continuous variables were assessed using Spearman test. For all tests, p-value ≤ 0.05 was considered significant.

Moreover, we evaluated the KORQ psychometric properties using WINSTEPS (version 3.92.1, Chicago, IL, USA; www.winsteps.com)^21^. Person and item measures were also examined in a Rasch model using infit and outfit item statistics. To test the hypothesis that the KORQ measures a single underlying construct, we initially evaluated the fit statistics, recording them as mean square standardized residuals (MNSQ); The fit of the Rasch model was evaluated with the infit and outfit statistics. Values between 0.7 and 1.3 are considered acceptable for MNSQ values of infit and outfit^22,23^.

## Results

A total of 100 keratoconus patients with a median age of 27.47 ± 7.02 years (range 18–51 years) were included in this cross-sectional registry-based study. The mean value of K max was 53.65 ± 5.00 (D) in the best eye and 59.67 ± 7.52 (D) in the worst eye. Additionally, the mean BCVA was 0.31 ± 0.29 and 0.68 ± 0.40 (LogMAR) in the better and worst eye, respectively; 67% of patients reported itching eyes, but only 31% reported use of medication; 26% wore rigid contact lens; 26% underwent crosslinking in at least one eye; 7% underwent intrastromal corneal ring procedure; and 8% underwent penetrating corneal transplantation. There were no statistically significant differences between the male and female subgroups, in relation to the keratoconus staging, hydrops or treatments performed. However, the males in our study had slightly better visual acuity (0.26 ± 0.29 vs 0.36 ± 0.29, *p* = 0.0451). Table 3 shows the study patients’ demographic and clinical variables.

**Table 3:** Demographic characteristics and clinical variables of keratoconus patients.

Parameter	Value
Mean age (years-old), ± SD	27.47 ± 7.02
Sex (male/female)	45/55
**Visual Acuity (LOGMAR), mean ± SD **	
Better eye	0.31 ± 0.29
Worse eye	0.68 ± 0.40
**Maximum keratometry (D), mean ± SD**	
Better eye	53.64 ± 5.00
Worse eye	59.67 ± 7.52
**Corneal curvature steep K (D), mean ± SD**	
Better eye	49.44 ± 4.23
Worse eye	53.96 ± 6.28
**Pachymetry (mean, microns) ± SD**	
Better eye	462.86 ± 49.48
Worse eye	433.27 ± 69.13
**Stage (1,2 or 3; better eye), n**	
1	15 (15.00)
2	46 (46.00)
3	39 (39.00)
**Stage (1, 2 or 3; worse eye), n**	
1	4 (4.00)
2	26 (26.00)
3	70 (70.00)
Allergic conjunctivitis, n	67 (67.00)
Use eye allergy medication, n	31 (31.00)
Hydrops, n (%)	12 (12.00)
RGP contact lens wear, n	26 (26.00)
Crosslinking, n	26 (26.00)
Intraestromal corneal ring, n	7 (7.00)
Corneal transplant, n	8 (8.00)

*SD* Standard deviation; *D* Diopter; *RGP* rigid gas permeable.

The KORQ psychometric properties were evaluated using WINSTEPS (version 3.92.1) in a Rasch model which used infit and outfit item statistics. According to our analysis, we found that the item Q10 from the “Activity Limitation” scale and items Q2, Q4, Q5, Q9 and Q11 from the Symptoms scale were misfitted (Table 4). After excluding these items that were misfitted, we conducted a principal component analysis of the residuals (difference between the observed and expected responses) to investigate unidimensionality. Data were considered unidimensional if most of the variance was explained by the principal component, whitout significant explanation of the residual variance in contrast to the principal component. The raw variance explained by measures was 66.6% and 56.9% in the Activity Limitation and Symptoms scales, respectively. We also found that the unexplained variance in 1^st^ contrast was 2.17 and 2.27 eigenvalues in both scales, respectively. Despite this, we found that the disattenuated person-measure correlation in the first contrast was > 0.50, suggesting that clusters belonged to random noise regardless of the multidimensionality issue. The person separation index, which is a measure of how broadly people can be distinguished into statistically distinct levels, is defined as the ratio of the variance in the person measures for the sample to the average error in estimating these measures. It is a measure of how broadly the persons could be distinguished into statistically distinct levels. The person separation reliability coefficient describes the reliability of the scale to discriminate between the persons with different abilities. Thus, a person separation index of ≥ 2.0 or a reliability value of ≥ 0.8 represents the minimum acceptable level of separation^24^. We found a person separation index of 3.84 and 2.46 in the Activity Limitation and Symptoms scales, respectively. Moreover, a person reliability value of 0.94 and 0.86 was found for both scales, respectively. This analysis provides information that the Portuguese translated KORQ is a unidimensional and psychometrically valid tool for assessing QoL in keratoconus patients.

**Table 4:** Fit Statistics using Rasch Analysis with respective Subscales from Keratoconus Outcomes Research Questionnaire (KORQ).

Questions	Subscale	Measure	Infit MNSQ	Outfit MNSQ
Q1	Activity limitation	− 0.27	0.93	1.01
Q2		0.41	0.79	0.81
Q3		− 1.36	0.98	0.88
Q4		− 0.20	1.02	1.11
Q5		0.30	0.75	0.83
Q6		1.54	1.14	0.97
Q7		1.48	0.99	1.02
Q8		0.92	0.93	0.85
Q9		− 2.31	0.86	0.80
Q10		− 1.13	1.44	2.40
Q11		1.66	0.95	0.86
Q12		0.98	1.05	1.00
Q13		− 0.78	1.38	1.39
Q14		− 0.67	1.13	1.10
Q15		1.84	1.04	0.89
Q16		− 0.10	0.77	0.78
Q17		− 2.01	0.97	0.83
Q18		− 0.30	0.83	0.85
Q1	Symptoms	− 0.95	0.91	0.81
Q2		0.78	1.35	1.27
Q3		0.28	0.99	1.21
Q4		− 0.73	1.91	2.42
Q5		0.64	1.59	1.62
Q6		0.28	0.98	0.96
Q7		0.30	0.91	0.92
Q8		0.13	0.95	0.94
Q9		0.27	0.49	0.49
Q10		− 0.75	0.81	0.95
Q11		− 0.26	0.62	0.62

*MNSQ* mean square.

We used the final score obtained from the activity limitation and symptom questionnaire after excluding the misfitted items based on our Rasch analysis. Table 5 further presents the KORQ scores according to the clinical and demographic parameters. Univariate analysis showed no statistically significant differences in functional and symptom score values according to age, steep keratometry, pachymetry, clinical and surgical treatments, and stage and type of treatment (*p* > 0.05). However, a statistically significant association was observed between gender and functional scores (*p* = 0.01), with men having lower scores than women, but without statistically significant differences in the symptom scores (*p* = 0.07). Moreover, a statistically significant association between visual acuity and functional (*p* < 0.001) and symptom (*p* < 0.001) scores was also observed, wherein individuals with BCVA < 0.3 LogMAR showed lower scores than individuals with BCVA ≥ 0.3 LogMAR.

**Table 5:** Univariate analysis on the association between clinical and demographic factors and Functional and Symptoms scores on the KORQ Questionnaire.

Variable	Functional score		Symptoms score	
	*mean* ± *sd*	*p value*	*mean* ± *sd*	*p value*
Sex				
Female	43.22 ± 20.14	0.01	59.75 ± 19.74	0.07
Male	34.48 ± 18.98		52.78 ± 19.01	
Age				
≤ 27	36.60 ± 17.54	0.31	53.09 ± 18.15	0.12
> 27	42.56 ± 22.44		60.93 ± 20.69	
Allergic conjunctivitis				
Yes	39.33 ± 20.21	0.94	57.54 ± 18.79	0.58
No	39.19 ± 19.91		54.73 ± 21.41	
Visual acuity (better eye, in LOGMAR)				
≥ 0.3	48.63 ± 19.10	< 0.01	63.94 ± 16.72	< 0.01
< 0.3	29.56 ± 16.01		48.99 ± 19.68	
Corneal curvature steep K (better eye)				
< 52	35.80 ± 16.24	0.24	52.36 ± 19.03	0.16
≥ 52	41.08 ± 21.60		58.81 ± 19.71	
Pachymetry (better eye)				
> 450	39.24 ± 18.21	0.81	56.45 ± 18.58	0.74
≤ 450	39.38 ± 23.27		56.91 ± 21.72	
Stage better eye				
1	34.92 ± 17.59	0.63	53.15 ± 17.64	0.32
2	40.43 ± 18.77		54.45 ± 18.94	
3	39.62 ± 22.42		60.51 ± 20.83	
Treatment				
No treatment / spectacles	40.73 ± 19.58	0.36	55.56 ± 18.39	0.91
RGP/ICRS/CXL/PKP	37.96 ± 20.50		57.59 ± 20.83	
Treatment				
No surgical treatments	40.37 ± 20.05	0.44	56.18 ± 19.68	0.88
Surgical treatments	37.44 ± 20.08		57.35 ± 19.79	
RGP contact lens				
Yes	36.60 ± 21.36	0.45	57.40 ± 22.79	0.79
No	40.23 ± 19.58		56.34 ± 18.55	
Crosslinking				
Yes	33.22 ± 17.20	0.07	54.91 ± 18.02	0.59
No	41.42 ± 20.59		57.22 ± 20.24	
Intraestromal corneal ring				
Yes	51.32 ± 15.90	0.18	64.68 ± 21.06	0.20
No	38.38 ± 19.32		56.01 ± 19.50	
Penetrating keratoplasty				
Yes	43.84 ± 15.55	0.31	62.63 ± 20.43	0.39
No	38.89 ± 20.37		56.09 ± 19.58	
RGP contact lens	39.23 ± 22.17	0.39	58.16 ± 23.95	
ICRS	49.00 ± 32.10		62.42 ± 25.31	0.71
CXL	32.90 ± 17.71		54.54 ± 18.73	
Corneal Transplant	43.84 ± 15.55		62.63 ± 20.43	
Hydrops				
Yes	40.15 ± 17.50	0.71	61.15 ± 18.46	0.36
No	39.17 ± 20.42		56.00 ± 19.80	
General	39.29 ± 20.01		56.62 ± 19.63	

*CXL* corneal collagen cross-linking; *RGP* rigid permeable gas; *ICRS* intraestromal corneal rings; *PKP* penetrating keratoplasty; *K* keratometry; Data are mean ± standard deviation, unless otherwise indicated.

**Figure 1:**
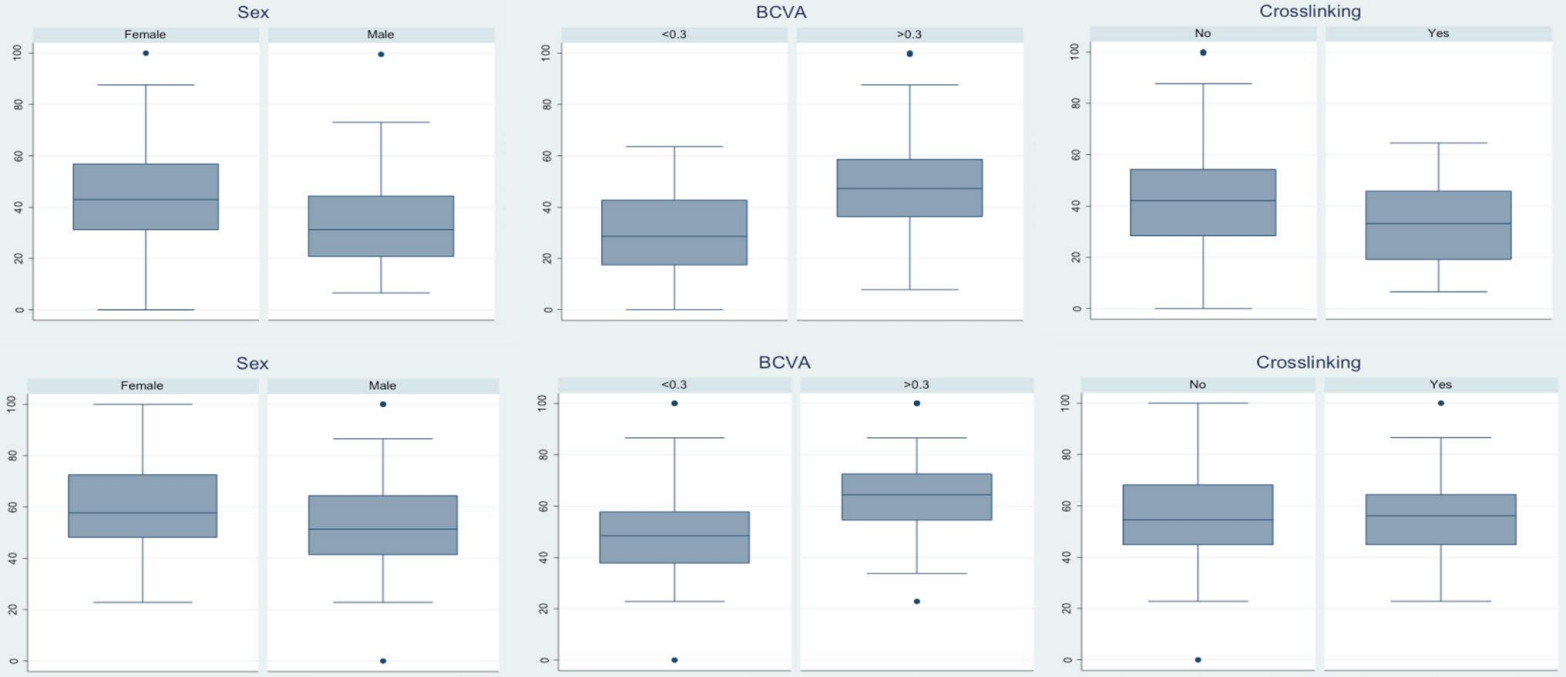
Above, boxplots functional score according to sex (*p* > 0.05), BCVA (*p* < 0.001) and crosslinking (*p* = 0.02). Below, boxplots symptom score according to sex (*p* > 0.05), BCVA (*p* < 0.001) and crosslinking (*p* > 0.05).

Table 6 further shows the results of the multiple linear regression for functional and symptom scores, in which statistically significant associations were observed between functional score, visual acuity, and history of crosslinking. Individuals with BCVA < 0.3 LogMAR had an average score of 18.39, which was lower than those with BCVA ≥ 0.3 LogMAR, and individuals with a history of crosslinking had an average score of 9.12, which was lower than those who had no such history. Regarding symptom score, only visual acuity was associated with the final score, wherein individuals with BCVA < 0.3 LogMAR showed an average score of 14.29, which was lower than those with BCVA ≥ 0.3 LogMAR. These results are represented more in detail by a boxplot in Fig. 1.

**Table 6:** Multiple linear regression to functional and symptoms scores according to stepwise backwards modeling.

	Multiple linear regression Function score		Multiple linear regression Symptoms score	
	*Coefficient (95%IC)*	*p*	*Coefficient (95%IC)*	*P*
**Gender**				
Female	Reference	–	Reference	–
Male	− 6.07 (− 12.99 a 0.85)	0.085	− 4.78 (− 12.14 a 2.58)	0.200
**Visual Acuity (better eye, in LOGMAR)**				
> 0.3	Reference	–	Reference	–
≤ 0.3	− 18.39 (− 25.27 a − 11.50)	** < 0.001**	− 14.29 (− 21.61 a − 6.96)	** < 0.001**
**Crosslinking**				
No	Reference	–	Reference	–
Yes	− 9.12 (− 16.88 a − 1.37)	**0.022**	− 3.03 (− 11.28 a 5.22)	0.468

**Figure 2:**
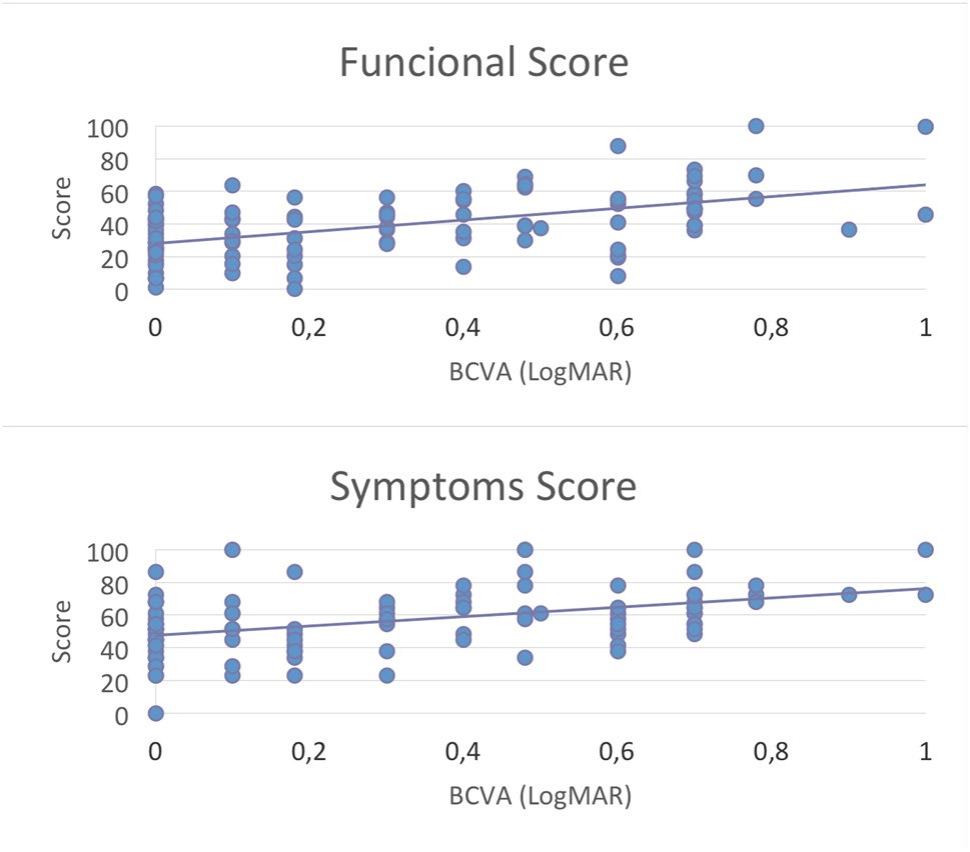
Spearman correlation test showed a positive correlation between visual acuity in LogMAR and both functional (r = 0.4934, *p* < 0.001) and symptoms (r = 0.4457, *p* < 0.0001) scores.

Spearman correlation test showed a positive correlation between visual acuity in LogMAR and both functional (r = 0.4934, *p* < 0.0001) and symptoms (r = 0.4457, *p* < 0.0001) scores. Figure 2 illustrates these results.

## Discussion

Most of the existing questionnaires are first generation questionnaires based on classical test theory, which uses summary scoring. This type of scoring is an overly simplistic method, wherein all items in a scale or a questionnaire are assumed to have an equal weight, and the response options are located at equal distances. Furthermore, it falsely assumes categorical ordinal data as interval-level data^17^. Modern psychometric methods, such as Rasch analysis, on the other hand, can convert categorical data into interval-level data using logarithmic transformation^25^. Additionally, Rasch analysis can use Andrich rating-scale and Partial-credit models to obtain the estimates of each item`s required ability, each subject`s perceived ability and each response category’s thresholds^25^. In this study we evaluated the psychometric properties of KORQ using an Andrich rating scale model, corroborating previous studies showing that KORQ is a psychometrically robust PRO measure to evaluate QoL parameters in individuals with keratoconus^15,16^. In this study, a validated version of the KORQ was applied in a large sample of keratoconus patients and the data presented herein represent the first cohort of patients evaluated using the KORQ tool.

In this sample, there was less impact in the activity limitation and symptoms of those patients with better vision (visual acuity < 0.3 LogMAR). We used the BCVA of the best-seeing eye based on previous studies that observed that ability to perform vision-related activities of daily living seems to be primarily a function of the vision in the best eye^26–28^. Additionally, QoL decline with worse visual acuity parallels findings of the Collaborative Longitudinal Evaluation of Keratoconus study, which similarly demonstrated that VA worse than 20/40 (decimal = 0.5) and corneal curvature ≥ 52 D were associated with significantly lower scores on all scales of the NEI-VFQ (National Eye Institute Visual Function Questionnaire)^29^. Moreover, in accordance with our findings, Gothwal et al.reported no statistically significant difference in the vision-specific functioning or emotional well-being between moderate and severe keratoconus groups, concluding that QoL did not vary as a function of disease severity in their cohort^30^.

In our study, multivariate analysis demonstrated that a history of corneal crosslinking was the only intervention significantly associated with functional scores. Indeed, Cingu et al. demonstrated that corneal collagen cross-linking led to significant improvements in keratometric readings including Kmax, associated with improved VRQoL and lower trait anxiety^31^. Furthermore, Labiris et al.^32^, while investigating the effects of CXL treatment on QoL of KC patients, found that dependency and mental health scores were reported to be better after CXL. They therefore concluded that CXL treatment had halted QoL deterioration of QoL in these patients, with evidence of gradual WoL worsening in untreated KC patients. This reinforces the importance of this procedure, given its benefits in limiting keratoconus progression, whicj not only reduces the need for transplantation^33^, but also apparently improves QoL. However, it is difficult to establish which factors that lead patients with a history of crosslinking to have better QoL scores, in addition to those previously mentioned, as well as to establish a causal relationship between the procedure QoL improvement. For that, it would be necessary a longitudinal study, with pre and post procedure evaluation, which is not the design of our study.

Despite these findings, our study had certain limitations. First, patients with keratoconus were recruited from a single tertiary eye hospital, thus only a few patients had early stages of the disease. Therefore, this cohort may not be representative of the Brazilian population. Our results should be confirmed through future studies with a larger population, especially with patients in earlier stages of the disease, which may also provide new findings. Second, this was a cross-sectional QoL study in keratoconus patients. We could only report associations between clinical parameters and QoL scores, but could not establish any causative relationships in the absence of longitudinal evaluation. Additionally, despite the KORQ having excellent psychometric properties as the currently recommended questionnaire to measure keratoconus outcomes, it only measures two QoL domains: activity limitation and symptoms. Items on other QoL domains, including psychosocial well-being and inconveniences, are not yet included in the KORQ.

Even with these limitations, our study had several potential implications. To our knowledge, and to date, this is the first study to apply KORQ to a group of keratoconus patients. This study has shown that better visual acuity is the main variable associated with QoL scores, which is in agreement with findings from other large case series, using distinct tools^3,26,29^. Therefore, improving visual acuity should be a constant goal in the treatment of keratoconus through any currently available option according to disease stage and improvement rate. Among these, corneal CXL is a well-known procedure to stabilize keratoconus progression. We observed that CXL may have a significant impact on patients’ QoL. Additionally, we also found that only a few patients were receiving medication for allergic conjunctivitis (31%), despite the higher number of related symptoms reported (67%). Considering that continuous corneal micro trauma is an important and well-known risk factor for disease progression, it is mandatory that all patients receive education about allergy signs and symptoms to prompt recognition and treatment of acute episodes, incorporating preventive measures in daily life.

In conclusion, our findings show that BCVA in the better eye and history of crosslinking are factors associated with higher QoL scores using the KORQ. Strategies to prevent keratoconus from reaching severe stages and, consequently, worse visual acuity, are warranted to maintain QoL. Thus, rehabilitation strategies focusing on reading and mobility issues are needed to improve QoL in those who already managing severe diseases.

## Data Availability

The data that support the findings of this study are available from the corresponding author, RDPP, upon request.
